# The Mediating Role of Compassion between Social Job Resources, and Healthy Healthcare Professionals: A Cross-Sectional Study with Gender Perspective

**DOI:** 10.3390/ijerph19127500

**Published:** 2022-06-19

**Authors:** Mabel San Román-Niaves, Cristián Coo, Susana Llorens, Marisa Salanova

**Affiliations:** WANT Research Team, Universitat Jaume I, 12071 Castelló de la Plana, Spain; coo@uji.es (C.C.); llorgum@uji.es (S.L.); salanova@uji.es (M.S.)

**Keywords:** compassion, healthcare context, social resources, well-being, healthy employees, healthy outcomes

## Abstract

The aim of this study is to examine the role of Compassion towards others as a mediator between Social Job Resources (social support climate, coordination, and positive leadership), Healthy Employees (psychological well-being such as resilience, engagement, and optimism) and Healthy Organisational Outcomes (in-role performance, extra-role performance and commitment) from a gender perspective in healthcare professionals. Through the multiple analyses of variance, structural equation models, and multiple-group analyses in a sample of 1420 healthcare professionals from different public and private hospitals in Spain, this study proved the existence of gender differences, with women perceiving higher levels of Compassion. Moreover, this study shows that Compassion partially mediates the relationship between Social Job Resources and Healthy Employees. In addition, Compassion partially mediates the relationship between Social Job Resources and Healthy Organisational Outcomes. Finally, Healthy Employees mediate the positive relationship between Social Job Resources and Healthy Organisational Outcomes. This is an innovative contribution to the limited research examining Compassion towards others as a personal resource that can have a positive impact in the workplace. The results also propose a way to develop and conduct interventions in order to increase Compassion towards others in the healthcare context.

## 1. Introduction

The aim of this study is to examine the role of Compassion as a mediator between Social Job Resources (social support climate, coordination, and positive leadership), Healthy Employees (psychological well-being such as resilience, engagement, and optimism) and Healthy Organisational Outcomes (in-role performance, extra-role performance and commitment) from a gender perspective in healthcare professionals.

The need for Compassion towards others has reached an unprecedented level during our time and bears no contextual comparison, particularly among health care professionals (HCPs), who are currently facing consistently stressful situations. These situations expose them to multiple psychosocial risks at work, such as quantitative overload, role stress, exhaustion, mortal anguish (HCPs know what to do, but cannot act), lack of companionship and lack of time [1]. Moreover, inadequate staffing levels and longer shifts (i.e., more than 12 h [2]) hinder clinicians’ ability to establish therapeutic relationships with patients. This, in turn, increases the workload, causing stress and exhaustion [3]. Additionally, HCPs are under increasing pressure to meet performance targets and achieve “more with less” service efficiencies [4]. All these negative outcomes have a serious impact on the health of patients and perception of the quality of care they receive [5].

Research suggests that situations of deterioration of mental health, motivation and well-being can be alleviated by increasing job and personal resources [6]. For example, in the Job Demands and Resources (JD-R) Model, Bakker and Demerouti [7] indicated the importance of the personal resources, which diminish the negative impact of job demands and enhance the positive impact of job demands on motivation, particularly on the challenges that may arise during the working day.

Personal resources are defined as psychological characteristics related to resilience and to the ability to control and to positively impact one’s own environment, which help workers to achieve their goals and encourage personal and professional growth [6]. Research has proved the importance of personal resources as mediators in the relationship between job resources and well-being, including self-efficacy, optimism, and organisational self-esteem [8] or psychological capital (PsyCap) (hope, resilience self-efficacy and optimism) [9]. Employees with high levels of PsyCap perceive fewer job demands and bring higher job resources [8]; they therefore feel less exhausted and are more vigorous, which is directly related to engagement [10].

Compassion is one of the personal resources that research has proved to be relevant for HCPs [11]. Gilbert and Choden [12] defined Compassion “as a motivation that orientates to a sensitivity to suffering in self and others with a commitment to try to alleviate and prevent it”. Research has established that Compassion towards others can help HCPs increase their self-esteem and appreciation for their work and to provide a high-quality care service [13]. Moreover, Compassion towards others can help to reduce perceived stress, anxiety, depression, burnout and improve emotion regulation [14].

So far, Compassion looks like an effective driver to improve wellbeing and could be a personal resource relevant to HCPs to cope with job stress and contribute to healthy and positive healthcare organisations. In that sense, Salanova et al. [15,16] proposed that Healthy and Resilient Organisations (HEROs) are “those organisations that promote healthy resources and practices” and “have healthy employees and workgroups that enjoy high psychosocial well-being, which in turn is related to healthy organisational results”. The HERO model [15] considers a motivational process in which Social Job Resources are vital to face job demands, leading to Healthy Organisational Outcomes. The HERO model explains that, in situations of excessive demands, personal and social resources must be present in order to prevent a deterioration in health, motivation, and performance. Compassion towards others can be considered as a personal resource that positively affects the three variables of the HERO model, i.e., organisational resources and practices, healthy employees, and healthy organisational outcomes.

To this end, there is evidence about the positive effects (i.e., reducing job stress and improving wellbeing) of cultivating Compassion towards others, particularly considering the gender perspective. As mentioned earlier, Compassion is a process of alleviating suffering and caring for others, and this definition is often seen as gendered since prescriptive gender roles see it more as a feminine trait [17]. There is limited research regarding the effects of gender on Compassion, so this research provides a great opportunity to show the role of Compassion from a gender perspective.

There is not much research on the premises and consequences of developing Compassion in the healthcare context. For this reason, it is important to explore the role of Compassion as a personal resource that could be developed by specific job resources that, in turn, can promote mental health, well-being (Healthy Employees) and positive results. In addition, the importance of studying Social Job Resources must also be highlighted, as they play a fundamental role in shaping employees’ experiences and behaviours [18].

To address this issue, this article aims to examine the extent to which Compassion, considering gender, mediates between Social Job Resources (social support climate, coordination, and positive leadership) and Healthy Employees (engagement, resilience, and optimism) that, in turn have a positive effect on Healthy Organisational Outcomes (commitment, in-role, and out-of-role performance).

## 2. Theoretical Framework

### 2.1. Compassion towards Others as a Personal Resource

In recent years, interest in studying Compassion as a personal resource has increased [19]. Despite this interest, there is a lack of research emphasising its role as a personal resource and how it can make job resources available in the healthcare context for HCPs. Compassion in medical care has received increasing attention from the health promotion literature, health care policy and professional organisations in recent years [20], and interest in Compassion towards others has also increased due to its positive impact on the health of patients [21].

Although the link between high quality care and Compassion is often assumed to be part of the job characteristics of the HCPs [22,23], and most HCPs are dedicated to performing their practice with Compassion, incidents of poor care have raised international concern about the state of Compassion in healthcare systems [20].

In healthcare, Compassion towards others consists mainly of two elements: a deep awareness and willingness to gain knowledge of people’s suffering and the desire to alleviate suffering [24]. One of the most important characteristics that differentiates Compassion towards others from other prosocial behaviors, like empathy and pity, is the powerful desire to alleviate suffering [20]. In addition to fulfilling a professional duty, research increasingly suggests that HCPs’ capacity to be compassionate is associated with better outcomes, including patient satisfaction, better quality doctor-patient interactions, and better long-term patient outcomes, both psychically and psychologically [25].

HCPs select treatments based on their efficacy, sometimes to the disadvantage of the quality of human relationships. However, this does not appear to be what patients want, quite the opposite. They want to be treated as people, to talk about their situation [26] and to speak as openly as possible [27]. When a person is ill, they become particularly vulnerable and may require the assistance of others, even for the most insignificant things. In these situations, they want to be treated with care and Compassion [28]. Sinclair et al. [5] developed a “Compassion Empirical Model” where they explained the point of view of patients and family members regarding their needs for their HCPs’ compassionate skills. The results showed that communication, virtuous response and attending to needs are fundamental in HCPs to provide a quality service to their patients.

It is important to highlight the positive aspects of Compassion in HCPs mental health. Sansó et al. [29] implemented the Mindful-Based Stress Reduction Training (MBSRT) and Compassion Cultivation Training (CCT) in HCPs and the results of this training showed an improvement of their quality of life (Compassion, satisfaction, resilience, empathy and decreased burnout and Compassion fatigue). Other studies revealed that mind-body skills (MBS) and CCT diminished stress and improved mindfulness, empathy, and resilience [30,31]. Considering the above, it is important to study Compassion as a positive personal resource that can help to develop skills such as: knowing the patient, perceiving the patient’s suffering, identifying with and being kind to the patient and showing respect, among others [11].

Previous research has shown that there are significant gender differences in Compassion, with most studies showing that women have higher levels of Compassion [32,33,34,35,36]. This is an expected result as it may be related to women’s emotional structure and maternal instinct [37]. Currently, more than 75% of the working population in the health sector are women [38], which means that it is a feminised profession, as statistically the percentage of women compared to men is about 55%. Taking note of previous research, we are interested in how the gender variable can be linked to Compassion towards others.

### 2.2. The Mediating Role of Compassion towards Others between Social Job Resources and Healthy Employees

Compassion towards others is commonly seen as a fundamental social force that creates and enhances interpersonal relationships [39]. Likewise, it has been shown to improve well-being in vulnerable people, promoting an intimate bond between colleagues, and facilitating cooperation between strangers [40], thus engaging supportive behaviours [18]. People also feel Compassion for another person when they are in discomfort, distress and evident need and respond to this situation by helping them [41]. This prosocial behaviour helps to relieve the wounded individuals’ pain [42].

Social Job Resources are those aspects of work including the emotional and instrumental support of supervisors and work colleagues [43]. It could be expected that having a workplace climate with rich social relationships, social support, positive coordination among co-workers as well as a positive leadership will result in creating Compassion among HCPs. For example, it has been shown that social support gives the opportunity to the HCPs to receive feedback, to reflect, to share challenges, difficulties, and successes with colleagues, and to provide and receive support from leaders [44]. If leaders and managers create a positive and supportive environment for HCPs, the HCPs in question create a caring, supportive climate and give a higher quality care service [45]. Additionally, the coordination with HCPs’ colleagues creates important bonds and encourages sharing of responsibilities, thus creating basis to reduce suffering [46]. Social Job Resources can help to increase Compassion towards others in a more efficient and reliable manner [47].

Following on from this, research has shown that Compassion positively relates to indicators of psychological well-being, such as engagement, resilience, and optimism. Engagement can be defined as a positive, pleasing, job-related state of mind that is distinguished by vigour, dedication, and absorption [48]. In the healthcare context, it has been shown that Compassion is highly related to work engagement, particularly when the job tasks are very challenging [49], and HCPs who are Compassionate feel more engaged [50]. With regards to resilience, defined by Sutcliffe and Vogus [51] as “the maintenance of positive adjustment under challenging situations, so that the organisations emerge from those conditions strengthened and more resourceful”, if HCPs have high levels of Compassion, they are more likely to be more resilient, emerging stronger from adverse situations of crisis and trauma [52,53]. Finally, optimism is an individual’s tendency to believe that good things will happen to them [54]. Vogus et al. [47] showed that Compassion towards others is related to optimism as a positive response that delivers and re-establishes positive meaning and psychological well-being. These studies suggest that Compassion can lead HCPs to feel better (well-being), enjoy higher levels of mental health and, therefore, be more productive, which translates into the happy-and-productive worker thesis [55]. This thesis proposes that people who are happier at work are more productive than those who are less happy, which implies that the higher the job and personal satisfaction, the better the performance. Therefore, people who are happier in and out of the work context will perform even better [56].

In summary, the literature proves the importance of Social Job Resources as enhancers of Compassion towards others and their positive impact, which, in turn, has a positive effect on different elements of employee wellbeing. In other words, Social Job Resources increase Compassion towards others [57,58], making work engagement, resilience, and optimism readily available and useful for increasing the likelihood of enhancing HCPs psychological well-being. The present study will focus on investigating Compassion as a positive psychology mechanism that could lead to psychological well-being (i.e., engagement, optimism, and resilience) and excellent organisational outcomes.

### 2.3. The Mediating Role of Compassion towards Others between Social Job Resources, Healthy Organisational Outcomes and Productive Workers

Compassion towards others allows Social Job Resources to be more easily accessed and deployed. As for creating resources, reinforcing certain shared beliefs and values, and the cultivation of relational skills [39], this fostering of acts of Compassion can produce employees that are not only more engaged, but also more productive [59].

Previous research suggests that Compassion towards others helps people to recover from suffering and painful situations, overcoming these circumstances, re-engaging and improving their job performance [60]. Furthermore, developing compassionate skills allows people to attain a better work-life balance, which is related to Healthy Organisational Outcomes [60] and has a positive impact on employees’ job performance [61].

Considering commitment as another Healthy Organisational Outcome, promoting commitment in the healthcare context is an effective strategy to give a sense of belonging in the organisation [62], increase retention, bring a better-quality care service and more importantly, conserve patient health along with a great commitment to patients [63]. HCPs who are highly committed to their place of work maintain friendly relationships with colleagues and achieve tasks based on the organisation [64]. Besides, Compassion also drives commitment among employees [65].

This study is also based on the theoretical framework of the HERO Model [15,16], this model has been tested in different organisational sectors, where the main results showed that HEROs (healthy and resilient organisations) can enhance personal resources at work such as trust [66], whereas horizontal and vertical trust is positively related to team commitment (vigour, dedication, and absorption). Salanova et al. [15] indicated that the development of resilience can have significant effects on job performance as well. It can be assumed that the efficient use of social resources and organisational practices leads to a positive outcome that improves the employees’ psychological well-being [16].

This can be seen in the study conducted by Farris [67], showing effectiveness in the healthcare context. The results of the study mention that if HCPs are satisfied and happy with their work and the work environment, they will be better able to develop their skills to perform their tasks and, as a result, an increase in productivity will follow.

### 2.4. The Current Study

The present study aims to evaluate the mediating role of Compassion between Social Job Resources (social support climate, coordination, positive leadership), Healthy Employees (engagement, resilience, optimism) and Healthy Organisational Outcomes (in-role and extra-role performance, commitment) from a gender perspective, using the data collected in different hospitals in Spain. More specifically, we explore Compassion as a positive psychological mechanism that can lead to high levels of well-being for healthcare workers and lead to successful organisational outcomes. On the other hand, we also examine the role of the Social Job Resources as Compassion enablers. All these issues are reflected in the following hypotheses:

**Hypothesis** **1** **(H1).** *Women show higher levels of Compassion towards others than men.*

**Hypothesis** **2** **(H2).** *Compassion partially mediates the positive relationship between Social Job Resources and Healthy Employees.*

**Hypothesis** **3** **(H3).** *Compassion partially mediates the positive relationship between Social Job Resources and Healthy Organisational Outcomes (in-role, extra-role performance and commitment).*

**Hypothesis** **4** **(H4).** *Healthy Employees mediates the positive relationship between Social Job Resources and Healthy Organisational Outcomes.*

## 3. Materials and Methods

### 3.1. Participants

The sample consisted of 1420 HCPs from different public and private hospitals in Spain. Mean age was 41.72 (SD = 10.77), average tenure time was 12.3 years (SD = 10.1), 78.67% (1117) were women and 21.33% (303) were men.

The procedure consisted of two parts: The first part, which was the contact with the hospitals, was carried out through a non-profit organisation that organises a yearly national competition to award hospitals and services with high levels of psychosocial well-being. The call for entries was launched via the NGO’s website and social media. Hospitals and services interested in participating registered and submitted a nomination in accordance with the competition rules. As a pre-requisite, registered participants had to have the approval of their hospital/department administration. Once the registration deadline had passed, data collection began for a period of two months. The second part was the data collection, it was carried out through the Healthy and Resilient Organisations (HERO) Questionnaire [15,16] that included 5 items of the Brief Compassion Scale [68,69], and other variables. The questionnaire was distributed among all the participants through an on-line link, encouraging them to participate voluntarily. Informed consent was obtained from each participant and data protection protocols were strictly followed according to existing GDPR regulations. The Ethics Committee of Jaume I University approved the study (CD/57/2020).

### 3.2. Instruments

- *Compassion towards others* was measured using a brief adaptation of the Compassion Scale [68,69]. Five items were chosen from the original scale (since, after conducting the analyses, they were the ones with the best factor scores), distributed between self-kindness, common humanity, mindfulness, and non-judgement/forgiveness. The items are scored on a seven-point frequency rating scale ranging from 0 (never) to 6 (always). Sample items include “If I see that someone is having difficulties, I try to help” (kindness), “I try not to judge others when they make mistakes or are wrong” (non-judgement/forgiveness), “I think everyone feels sad sometimes, it is part of being human” (common humanity), “I usually listen patiently when people tell me about their problems” (mindfulness). Cronbach’s Alpha (α = 0.65), and McDonald’s Omega (ω = 0.65): the Cronbach’s Alpha is moderate, so it is above the acceptable limit [70], which is why the decision was made not to remove the item that scored less, as it would remain with two of the three constructs that make up Compassion.
- *Social Job Resources* were measured using the HERO questionnaire subscale by the same name including social support climate, positive leadership, and coordination. Each of these items was represented by a single item, where each of these single items is the mean of the HERO subscale [15,16]. Participants answered using a seven-point frequency type scale with scores from 0 (never) to 6 (always). Sample items include “Degree to which your supervisor considers the needs of your service/care unit, recognising the effort and achievement of goals of the service/care unit” (positive leadership), “Degree to which you feel supported by your colleagues and supervisor personally and professionally” (social support climate) and “Degree to which you are coordinated with your work team, in order to respond to work situations” (coordination). (Cronbach’s Alpha (α = 0.80), and McDonald’s Omega (ω = 0.81))
- *Healthy Employees* were measured using the HERO questionnaire subscale by the same name including engagement, resilience, self-efficacy, and optimism. Each of these items was represented by a single item, where each of these single items is the mean of the HERO subscale [15,16]. Participants answered using a seven-point frequency with scores from 0 (never) to 6 (always). Sample items include “Degree to which you feel immersed, full of energy and dedicated to your work, creating a positive climate of fulfilment and hope” (engagement), “Degree to which you feel capable of emerging stronger after facing adversity and failures at work” (resilience), and “Degree to which you generally expect the best in difficult times, you are optimistic about the future and in general, you expect more good things to happen than bad” (optimism). (Cronbach’s alpha (α = 0.81), and McDonald’s omega (ω = 0.81))
- *Healthy Organisational Outcomes* were measured using items from the HERO questionnaire subscale by the same name including a single item each for commitment, extra-role, and in-role performance [15,16]. Each one of these elements was represented by a single item, this was due to factor loadings where they fitted best as observed variables. Participants answered using a seven-point frequency type scale with scores from 0 (never) to 6 (always). Sample items include “Degree to which your work tasks are carried out and fulfilled” (in-role performance), “Degree to which tasks that exceed what is prescribed by your work are performed” (extra-role performance), “Degree to which you feel committed to the health centre and its outcomes, how proud you feel to belong there” (commitment).

### 3.3. Data Analyses

First, to test the Hypothesis 1, Multiple Analyses of Variance (MANOVA) were performed with IBM SPSS Statistics 28.0 to test for significant differences in the study variables according to gender (women and men). For these analyses, the general database of 1420 HCPs (78.6% women and 21.4% men) was used. Subsequently, chi-square tests (χ^2^) and t-tests were performed, where significant differences in favour of women were found. Based on Yoon and Lai [71], who mention that groups that are very unbalanced can alter the results, the decision was made to randomise the sample to 606 HCPs (N = 303 men, N = 303 women). The random sample of women and men was performed using the R 4.1.0 sample function [72].

Considering that the database has a higher percentage of women, it was decided to use the women (N = 1117) to test the rest of the hypothesis, so descriptive and correlation analyses were the second step. Reliability was estimated with Cronbach’s alpha and McDonald’s hierarchical omega coefficients [73]. Afterwards, several different models were examined using a structural equation modelling approach (SEM) with maximum likelihood estimation in SPSS AMOS 26. To establish goodness of fit we calculated relative and absolute fit-indexes, specifically, chi-squared (χ^2^) and normed chi-squared (χ^2^/df), root–mean–squared error of approximation (RMSEA) with upper and lower confidence intervals, Tucker Lewis Index (TLI), and Comparative Fit Index (CFI) following the cut-off points suggested by Schreiber [74]. We also calculated the indirect effects of the different mediation paths, their statistical significance and confidence intervals using R package for causal mediation analysis [75].

To test our proposed hypotheses, we established six different SEM models of increasing complexity with the female sample. For Hypothesis 2, we first established Model 1 (M1) where we tested the partial mediating role of Compassion towards others between Social Job Resources and Healthy Employees. Model 2 (M2) tested the full mediating role of Compassion towards others between Social Job Resources and Healthy Employees as an alternative model to M1.

Next, to test Hypothesis 3 we established Model 3 (M3) which was built upon M1 and extended it including the partial mediating role of Compassion towards others between Social Job Resources and Healthy Organisational Outcomes. In Model 4 (M4) we tested the full mediation of Compassion towards others between Social Job Resources and Healthy Organisational Outcomes as an alternative model.

**Figure 1:**
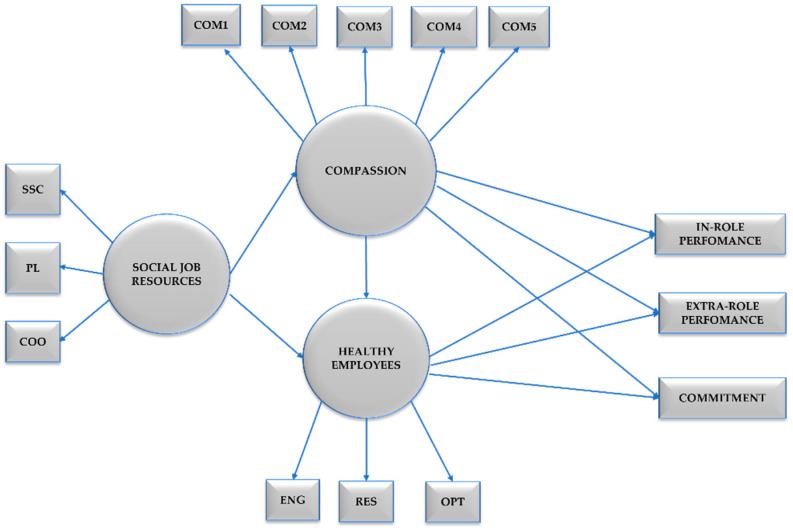
Research model involving Compassion, Social Job Resources, Healthy Employees and Healthy Organisational Outcomes. Note. SSC = Social Support Climate, PL = Positive Leadership, COO = Coordination, COM1 = Compassion Item 1, COM2 = Compassion Item 2, COM3 = Compassion Item 3, COM4 = Compassion Item 4, COM5 = Compassion Item 5, ENG = Engagement, RES = Resilience, OPT = Optimism.

Additionally, to test Hypothesis 4 we established Model 5 (M5) which extended M3 including the partial mediation of Healthy Employees between Social Job Resources and Healthy Organisational Outcomes. Additionally, we tested in Model 6 the full mediation of Healthy Employees between Social Job Resources and Healthy Organisational Outcomes as an alternative model. Compassion towards others, Social Job Resources and Healthy Employees were included as latent variables Healthy Organisational Outcomes were included as three distinct observed variables, namely: in role performance, extra role performance, and organisational commitment (see Figure 1).

Finally, using the randomised sample of men and women (w = 303, m = 303), structural equation modelling analyses (SEM) were performed using multi-group analysis with SPSS AMOS 26.0 software to test the hypothesised model that assumes that Compassion partially mediates between Social Job Resources, Healthy Employees, and Healthy Organisational Outcomes. To explore gender invariance—following Chen [76]—we tested for configural (i.e., same structure across groups), metric (i.e., same factor loadings across groups), and scalar invariance (i.e., same intercepts across groups) through multiple–group CFA.

## 4. Results

### 4.1. Multiple Analyses of Variance (MANOVA)

First, the MANOVA was performed. Using the random sample, regarding gender (as an independent variable), and the rest of the study variables (Social Job Resources, Compassion, Healthy Employees, and Healthy Organisational Outcomes) as dependent variables. The results showed significant differences between the HCPs of men and women [F (10,595) = 2.047, *p* < 0.05]. Women also showed significantly higher levels of Compassion [F (1,888) = 10.135, *p* < 0.05]; mean for women = 4.957, mean for men = 4.763; and positive leadership [F (1,593) = 4.840, *p* < 0.05]; mean for women = 4.851, mean for men = 4.620.

### 4.2. Descriptive Analyses

Second, Table 1 shows the participant’s socio-demographic information. Table 2 shows the means, standard deviations, and intercorrelations of all the variables included in the study (N = 1420), i.e., Compassion (the mean of the 5 items), Social Job Resources (social support, coordination, positive leadership), observed variables of Healthy Organisational Outcomes (in-role performance, extra-role performance, commitment) and healthy employees (work engagement, resilience, optimism). The results show that all variables correlate, except for extra-role performance which has no correlation with resilience, optimism, and Social Job Resources.

### 4.3. Structural Equation Modelling Analyses

In SEM tests for Hypothesis 2, M1 showed good fit indexes, χ^2^ = 153.688; df = 41; *p* < 0.001; RMSEA = 0.05; TLI = 0.964 and CFI = 0.973. On the other hand, M2 showed less than acceptable fit indexes, χ^2^ = 503.951; df = 42; *p* < 0.001; RMSEA = 0.099; TLI = 0.858 and CFI = 0.891. Moreover, the mediation effect was significant (β = 0.143, *p* < 0.001).

For Hypothesis 3 results for M3 showed poorer fit indexes, χ^2^ = 190.792; df = 40; *p* < 0.001; RMSEA = 0.058; TLI = 0.933 and CFI = 0.951. M4 showed acceptable fit indexes in comparison, χ^2^ = 328.533; df = 43; *p* < 0.001; RMSEA = 0.077; TLI = 0.882 and CFI = 0.907. Again, all three mediation effects were significant, specifically in-role performance (β = 0.187, *p* < 0.001), extra-role performance (β = 0.128, *p* < 0.001) and organisational commitment (β = 0.190, *p* < 0.001).

**Figure 2:**
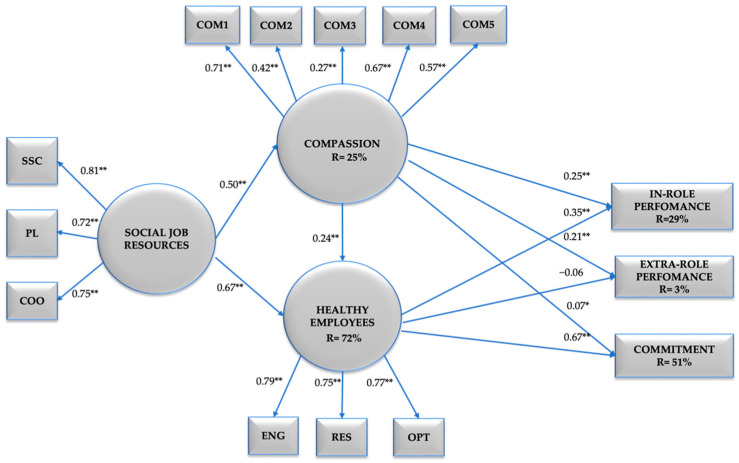
Final model: Structural Model of Compassion, Social Job Resources, Healthy Employees and Healthy Organisational Outcomes in women (N = 1117). Note. SSC = Social Support Climate, PL = Positive Leadership, COO = Coordination, COM1 = Compassion Item 1, COM2 = Compassion Item 2, COM3 = Compassion Item 3, COM4 = Compassion Item 4, COM5 = Compassion Item 5, ENG = Engagement, RES = Resilience, OPT = Optimism. ** *p* < 0.01, * *p* < 0.05.

Finally, in tests for Hypothesis 4, M5 showed acceptable fit indexes, χ^2^ = 268; df = 71; *p* < 0.001; RMSEA = 0.050; TLI = 0.952 and CFI = 0.963. In comparison, M6 showed poorer fit indexes, χ^2^ = 233.759; df = 68; *p* < 0.001; RMSEA = 0.047; TLI = 0.958 and CFI = 0.969. Once more, all three mediation effects were significant, specifically in-role performance (β = 0.235, *p* < 0.001), extra-role performance (β = 0.160, *p* < 0.01) and organisational commitment (β = 0.827, *p* < 0.001). A summary for the SEM models fit indexes is presented in Table 3. A diagram for the final SEM model derived from M5 is shown in Figure 2. The final model (M5) shows that Social Job Resources (i.e., social support climate, positive leadership, coordination) explain 23% (*p* < 0.01) of Compassion, and 72% (*p* < 0.01) of Healthy Employees (i.e., engagement, resilience, optimism) is explain by Compassion and Social Job Resources. Compassion and Healthy Employees explain 29% (*p* < 0.01) of in-role performance and 51% (*p* < 0.01) of commitment, while 30% (*p* < 0.01) of extra-role performance is explained by Compassion. See Figure 2.

### 4.4. Multiple-Group Configural Factor Analyses

Additionally, we conducted invariance analysis comparing the stability of the factor structure of our final SEM model (M5) between participants from different gender groups. Results are shown in Table 4.

**Figure 3:**
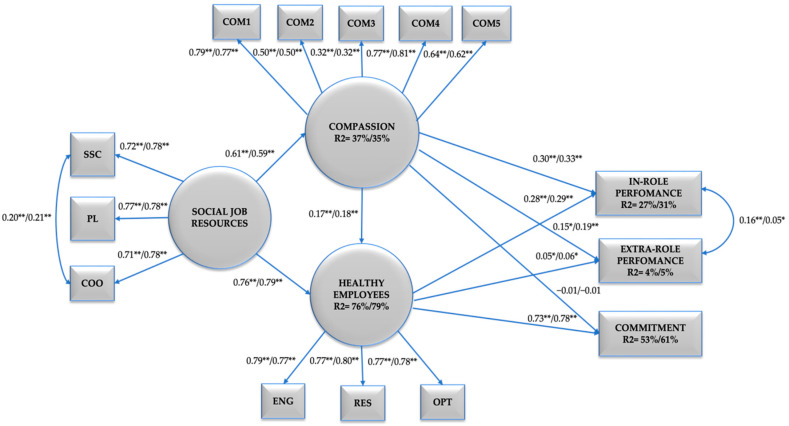
Final Model: Structural Model of Compassion, Social Job Resources, Healthy Employees and Healthy Organisational Outcomes in two samples, Women (N = 303) and Men (N = 303). Note. SSC = Social Support Climate, PL = Positive Leadership, COO = Coordination, COM1 = Compassion Item 1, COM2 = Compassion Item 2, COM3 = Compassion Item 3, COM4 = Compassion Item 4, COM5 = Compassion Item 5, ENG = Engagement, RES = Resilience, OPT = Optimism, IP = Intra-Role Performance, EP = Extra-Role Performance, COMM = Commitment. ** *p* < 0.01, * *p* < 0.05. The data on the left of the bar correspond to women and those on the right to men.

The baseline model (M5.1) showed adequate fit supporting configural invariance. Next, constraints were imposed on all factor loadings making them equal to examine metric invariance. The resulting model also showed adequate fit indexes (see M5.2). Comparison between M5.1 and M5.2 yielded a significant chi-squared difference test. As for the CFI and SRMR indexes, the differences were slightly above the 0.01 threshold for CFI and below for the SRMR; thus metric invariance was only partially supported. Since the criteria for metric invariance were not met, we did not test for stricter scalar invariance models. The results of this final multiple-group model are shown in Figure 3. For women, Social Job Resources (i.e., social support climate, positive leadership, coordination) explain 37% (*p* < 0.01) of Compassion, and 76% (*p* < 0.01) of Healthy Employees (i.e., engagement, resilience, optimism) is explained by Compassion and Social Job Resources. Compassion and Healthy Employees explain 27% (*p* < 0.01) of In-role Performance and 4% (*p* < 0.01) of Extra-role Performance, while 53% (*p* < 0.01) of Commitment is explained by Compassion. For men, Social Job Resources explain 35% (*p* < 0.01) of Compassion, and 79% (*p* < 0.01) of Healthy Employees is explained by Compassion and Social Job Resources. Compassion and Healthy Employees explain 31% (*p* < 0.01) of In-role Performance and 5% (*p* < 0.01) of Extra-role Performance, while Healthy Employees explain the 61% (*p* < 0.01) of Commitment. See Figure 3.

## 5. Discussion

The present study examined the role of Compassion towards others as a mediator between Social Job Resources, measures of employee mental health and performance, taking into account gender perspective in a sample of HCPs. The results suggest that women are more compassionate than men and, furthermore that Compassion towards others acts as a mediator between Social Job Resources such as coordination, positive leadership and social support climate, and workers’ mental health indicators such as work engagement, optimism, resilience, and performance just as in and extra role performance, and organisational commitment.

The current study makes an innovative contribution to the limited research examining Compassion towards others as a personal resource and mediator between Social Job Resources, Healthy Employees, and Healthy Organisational Outcomes. Based on the HERO model [15], we hypothesised and found that Compassion towards others has a positive relationship with Social Job Resources, Healthy Employees, and Healthy Organisational Outcomes. Moreover, it can be suggested that the happy-and-productive worker thesis [55] can also be reflected in the healthcare sector, where the higher the levels of Compassion, the greater the well-being, with better organisational results being a positive consequence.

The first hypothesis has been fulfilled, confirming that women are more compassionate that men. The results are in line with the studies conducted by Oruç et al. [33] and Arkan et al. [37], where the authors found that women, due to gender-specific characteristics (i.e., emotional structure and maternal spirit), tend to be more compassionate than men. Secondly, our second hypothesis reported that Compassion towards others partially mediates the positive relationship between Social Job Resources (social support climate, coordination, and positive leadership) and Healthy Employees (engagement, resilience, and optimism). Our study supports the statement by Kanov et al. [77] about Compassion being a mediator that benefits access and extends social resources that can be deployed in the workplace. This is clearly reflected in the model that we propose, where HCPs who are compassionate towards patients, relatives, and companions feel higher levels of Social Job Resources (i.e., social support climate, coordination, and positive leadership). Moreover, our results can be related to the study by Cosley et al. [53], where the authors found that Compassion increases the ability to make use of social support, and to the study conducted by Condon [78] that indicates that Compassion towards others increases prosocial behaviours.

Besides, our study also showed that Compassion towards others also mediates the relation between Social Job Resources and Healthy Organisational Outcomes (extra-role performance, in-role performance, and commitment) which confirms our third hypothesis. Our results also reinforce the statement by Lilius et al. [60], showing that Compassion towards others reinforces commitment to the organisation and reveals that a higher level of Compassion towards others increases job performance in the organisation, as well as in-role and extra-role performance.

Subsequently, regarding our fourth hypothesis, a replica of part of the relations proposed in the HERO model [15], the results confirmed the positive relationship between Social Job Resources, Healthy Employees, and Healthy Organisational Outcomes in the healthcare sector. This model proposes that the organisations that encourage healthy resources have also Healthy Employees and workgroups that enjoy high psychosocial well-being.

Finally, with the results obtained in the multi-group analyses, the first hypothesis was reaffirmed, showing that women do perceive higher levels of Compassion than men.

## 6. Conclusions

In conclusion, this study suggests the importance of developing social resources at work to increase Compassion for others, which can bring benefits to the well-being of HCPs, and can lead to them being happier and more productive at work. In addition, it can bring benefits to organisations as the study shows that they are more engaged, and their productivity can be higher. In addition, being compassionate leads them to better cope with work demands. Finally, differences in gender perspective have been found, showing that women tend to perceive higher levels of Compassion than men.

### 6.1. Theoretical Contributions

This article makes several contributions for our understanding of the role that Compassion towards others and the positive relationships it can have in the healthcare context with a gender perspective. Moreover, this study supports the HERO Model, incorporating for the first time the role of Compassion towards others as a personal resource and its impact on Healthy Employees (i.e., engagement, resilience, and optimism) and Healthy Organisational Outcomes (i.e., in-role and extra-role performance and commitment).

Secondly, this study showed that Compassion towards others increases happiness levels. This is an important contribution as happiness leads to many positive outcomes, such as positive mental health, and supports better relationships with co-workers [56,79]. It thereby improves productivity and engagement levels among colleagues [80,81]. This is also related to when compassionate behaviour is exhibited by the HCPs and supported by organisational processes, the organisation reaches higher levels of Healthy Organisational Outcomes.

Regarding the differences between gender, we analysed the differences between women and men, and the results demonstrated that women are more compassionate than men. Finally, we also tested the direct effect between Social Job Resources and Healthy Employees, and the results showed that Job Social Resources improve well-being (i.e., engagement, resilience, optimism) and provide the necessary tools to counter the job demands in the healthcare context.

### 6.2. Practical Implications

Though much importance has been given to the need of Compassion in healthcare in recent years [20], healthcare organisations should also be concerned with promoting their employees’ well-being, providing them with the tools and resources to face day-to-day situations. Situations related to the organisational structure may impede workers from profiting from specific job resources [6], which is why it is important to develop personal resources, such as Compassion towards others that, in addition to having recognition and gratitude for their work, helps employees to provide quality service to their patients [13]. Similarly, when patients feel that their clinician actively listens without interrupting, and is kind and generous, this certainly has a positive impact on the quality of care [82].

Based on the results of this study, we propose a way to develop and conduct interventions to increase Compassion in the healthcare context. For instance, increasing Compassion towards others as a personal resource not only has benefits for oneself but can help to transfer those resources into the workplace. Furthermore, Compassion is an important social resource in personal development that can increase the likelihood of prosocial behaviours that go beyond oneself and move towards others [83]. Aligned with our results, these conclusions suggest that interventions should focus on prosocial behaviours. In addition, this study showed that Compassion can be significantly affected by gender.

Lastly, this study shows that, while HCPs have higher levels of well-being at work, and the healthcare organisation enhances the Social Job Resources, this has a positive consequence on their job performance and on their commitment to the organisation.

### 6.3. Limitations and Future Research

Even though we obtained interesting results, the present study has numerous limitations. The first limitation is that the data is cross-sectional. Despite SEM analysis, precisely that proposed in M5, and while the model offers information about the probable direction of the relationships, the cross-sectional study does not allow us to identify or confirm predictive conclusions about the causal order of the variables studied. Future research should focus on performing longitudinal studies with the objective of discovering the causal order between the study variables.

Second, the Brief Compassion Scale [68,69] has a moderate Cronbach’s Alpha (α = 0.65) and is above the acceptable limit [70]. Furthermore, Loewenthal [84] suggests that for scales with less than 10 items, a score of 0.60 can be considered acceptable. This can be seen as a limitation as other authors disagree with this and consider 0.70 to be the acceptable limit [85,86]. In this study, if one of the lower scoring items is removed, the scale will be left without the dimension of common humanity, which is one of the constructs that make up Compassion. For this reason, it was decided to maintain the entire scale.

Third, currently there is a global health crisis caused by the Coronavirus Disease (COVID-19), that has resulted in HCPs being on the frontline for more than two years. This situation has generated high levels of stress, anxiety, and depressive symptoms. Barello and Graffigna [87] suggest that providing HCPs with tools to cope with such stressful situations can help them to remain resourceful and determined in the workplace. The data collection of this study was carried out in pre-pandemic times, where the workload surely had no comparison to what is being experienced now. That is why future research could be aimed to conduct studies on Compassion in times of COVID-19 to see the effects it has on both healthcare personnel and patients.

Fourth, the sample is mostly of female staff, who, as mentioned above, represent a high percentage in this sector. For future studies, the gender variable should be considered to assess the differential effects of gender and how this affects Compassion.

Fifth, this is a heterogeneous sample as it includes different HCPs from different departments, hospitals, and job positions. This may be a limitation, as it can be considered that the types of work performed in different positions, departments or services may vary from one another. However, despite these differences, HCPs work in a very similar context, where attention to or care for others (patients, relatives, and co-workers) is of great importance. This study reflects the organisation of an average hospital in Spain, where it could be considered that some job positions would need to have higher levels of compassion due to the type of activity they perform. For this reason, comparisons could be made between different services and professional profiles within the same hospital or different hospitals.

Sixth, this study looks at Compassion only in an interindividual way, that is, on a social level as prosocial behaviours. Future studies could focus on the group or team perspective considering the affective level and the relationships between peers. Moreover, it might be interesting to see how Compassion from a group perspective affects work demands (i.e., burnout).

Finally, due to the lack of studies that implement interventions in the healthcare context, we propose to carry out empirical studies testing the model with the variables outlined in this study in order to increase the levels of Compassion in HCPs.

## Figures and Tables

**Table 1:** Sociodemographic information of the participants (N = 1420).

Variables	Demographic Information
Age ranges	14.71% (209) aged 20–29 28.66% (407) aged 30–39 32.18% (457) aged 40–49 17.60% (250) aged 50–59 6.26% (89) aged 60–75
Job Position	39.64% (563) nurses 18.45% (262) nursing assistants 9.92% (141) physicians 9.71% (138) coordinators/supervisors 5.28% (75) dieticians and kitchen 3.87% (55) administrative 3.16% (45) technicians 9.92% (141) others (e.g., orderlies, psychologists, midwives, support staff)
Tenure	33.59% (477) one to five years 18.09% (257) six to 10 years 16.97% (241) 11 to 15 years 11.33% (161) 16 to 20 years 13.52% (192) 21 to 30 years 6.05% (86) 31 to 45 years
Type of contract	65.63% (932) permanent contract 22.6% (321) temporary contract 11.76% (167) another type of contract

**Table 2:** Descriptive statistics and correlations among all study variables.

	M	SD	1	2	3	4	5	6	7	8	9	10	11
1. Compassion	4.91	0.70	-										
2. Social Job Resources	4.89	0.83	−0.11 **	-									
3. Social Support	4.89	1.11	−0.12 **	−0.01	-								
4. Coordination	4.99	0.98	−0.62 **	−0.05	0.32 **	-							
5. Positive Leadership	4.82	1.24	−0.07 *	−0.02	0.28 **	0.83 **	-						
6. In-Role Performance	5.45	0.82	−0.09 **	−0.01	0.36 **	0.45 **	0.31 **	-					
7. Extra Role Performance	3.85	1.40	0.02	0.04	0.12 **	0.01	−0.00	0.05	-				
8.Organisational Commitment	4.96	1.14	−0.13 **	0.02	0.37 **	0.51 **	0.37 **	0.42 **	0.42 **	-			
9. Healthy Employees	4.66	0.94	−0.13 **	−0.01	0.43 **	0.72 **	0.58 **	0.60 **	0.56 **	0.43 **	-		
10. Work Engagement	4.81	1.01	−0.14 **	0.02	0.39 **	0.62 **	0.47 **	0.54 **	0.45 **	0.42 **	0.07 *	-	
11. Resilience	4.58	1.11	−0.11 **	−0.00	0.35 **	0.61 **	0.50 **	0.51 **	0.47 **	0.36 **	0.03	0.54 **	-
12. Optimism	4.58	1.21	−0.09 **	0.00	0.36 **	0.60 **	0.51 **	0.48 **	0.50 **	0.34 **	0.05	0.57 **	0.86 **

Note. M = Mean; SD = Standard Deviation. All scales were measured using a using a 7-point scale. N = 1420. ** *p* < 0.01, * *p* < 0.05.

**Table 3:** Goodness of fit indexes for tested SEM Models.

Model	χ²	df	χ²/df	*p*	TLI	CFI	RMSEA	Lower	Upper
M1 Partial Mediation	153.688	41	3.75	0.000	0.964	0.973	0.050	0.041	0.058
M2 Full Mediation	503.951	42	11.99	0.000	0.858	0.891	0.099	0.092	0.107
M3 Partial Mediation	190.792	40	4.769	0.000	0.933	0.951	0.058	0.050	0.067
M4 Full Mediation	328.533	43	7.640	0.000	0.882	0.907	0.077	0.069	0.085
M5 Partial Mediation	268.676	71	3.784	0.000	0.952	0.963	0.050	0.044	0.056
M6 Full Mediation	233.759	68	3.437	0.000	0.958	0.969	0.047	0.040	0.053

Note. χ^2^ = Chi-square; df = degrees of freedom; χ^2^/df = relative Chi-square; *p* = probability; TLI = Tucker–Lewis Index; CFI = Comparative Fit Index; RMSEA = Root Mean Square Error of Approximation.

**Table 4:** Fit indexes for single group and multi-group SEM Model 5.

	χ^2^	df	χ^2^/df	RMSEA	90% CI	CFI	TLI	SRMR	CMs	∆χ^2^ (∆ df)	∆CFI	∆SRMR
Single Group SEM												
M5	157.890 **	69	2.288	0.046	[0.037, 0.056]	0.973	0.964	0.032	-	-	-	-
Multiple Group (Gender)												
M5.1 configural invariance	337.390 **	186	1.814	0.037	[0.030, 0.043]	0.954	0.955	0.046	-	-	-	-
M5.2 metric invariance	380.419 **	187	2.034	0.041	[0.035, 0.047]	0.941	0.943	0.052	M5.1–M5.2	43.029 (1) **	0.013	0.006

Note. ** *p* < 0.001; χ^2^, Chi-square; df, degree of freedom; RMSEA, Root Mean Square Error of Approximation; CI, 90% confidence interval; CFI, comparative fit index; TLI = Tucker–Lewis Index; SRMR, Standardised Root Means Square Residual; CMs, Comparisons between Models.
